# A long way to go: a systematic review to assess the utilisation of sexual and reproductive health services during humanitarian crises

**DOI:** 10.1136/bmjgh-2017-000682

**Published:** 2018-05-03

**Authors:** Neha S Singh, Sarindi Aryasinghe, James Smith, Rajat Khosla, Lale Say, Karl Blanchet

**Affiliations:** 1 Health in Humanitarian Crises Centre, London School of Hygiene and Tropical Medicine, London, UK; 2 Centre for Maternal, Adolescent, Reproductive and Child Health (MARCH), London School of Hygiene and Tropical Medicine, London, UK; 3 Department of Reproductive Health and Research, World Health Organization, Geneva, Switzerland

**Keywords:** systematic review, health services research, maternal health, Hiv, other infection, disease, disorder, or injury

## Abstract

**Introduction:**

Women and girls are affected significantly in both sudden and slow-onset emergencies, and face multiple sexual and reproductive health (SRH) challenges in humanitarian crises contexts. There are an estimated 26 million women and girls of reproductive age living in humanitarian crises settings, all of whom need access to SRH information and services. This systematic review aimed to assess the utilisation of services of SRH interventions from the onset of emergencies in low- and middle-income countries.

**Methods:**

We searched for both quantitative and qualitative studies in peer-reviewed journals across the following four databases: EMBASE, Global Health, MEDLINE and PsychINFO from 1 January 1980 to 10 April 2017. Primary outcomes of interest included self-reported use and/or confirmed use of the Minimum Initial Service Package services and abortion services. Two authors independently extracted and analysed data from published papers on the effect of SRH interventions on a range of SRH care utilisation outcomes from the onset of emergencies, and used a narrative synthesis approach.

**Results:**

Of the 2404 identified citations, 23 studies met the inclusion criteria. 52.1% of the studies (n=12) used quasi-experimental study designs, which provided some statistical measure of difference between intervention and outcome. 39.1% of the studies (n=9) selected were graded as high quality, 39.1% moderate quality (n=9) and 17.4% low quality (n=4). Evidence of effectiveness in increasing service utilisation was available for the following interventions: peer-led and interpersonal education and mass media campaigns, community-based programming and three-tiered network of community-based reproductive and maternal health providers.

**Conclusions:**

Despite increased attention to SRH service provision in humanitarian crises settings, the evidence base is still very limited. More implementation research is required to identify interventions to increase utilisation of SRH services in diverse humanitarian crises settings and populations.

**Key questions**

**What is already known?**

- There are an estimated 32 million women and girls of reproductive age living in humanitarian crises settings, all of whom need sexual and reproductive health (SRH) information and services.
- Previous studies yielded mixed evidence for the implementation of SRH services in humanitarian crises.
- There has been no peer-reviewed study since 2004 that has comprehensively evaluated all pillars of the Minimum Initial Service Package for SRH in humanitarian crises settings.

**What are the new findings?**

- Interpersonal and peer-led education and mass media campaigns are effective interventions for increasing service utilisation of HIV, sexually transmitted infection and maternal health services in crises settings.
- Training lower cadre health workers from refugee or internally displaced populations to provide SRH services in crises settings can be effective in increasing SRH service use.

**What do the new findings imply?**

- The limited evidence base for SRH interventions highlights the need for improved research on the utilisation of SRH interventions in humanitarian crises.
- Rigorous implementation research is needed across a range of diverse crisis settings and populations to identify evidence-based interventions to increase the utilisation of SRH services.

## Introduction

Sexual and reproductive health (SRH) and rights are fundamental to individual health and well-being, as well as population health and development. Significant gaps in access to SRH information and services exist globally, which threaten the lives and well-being of individuals and their families. Unmet need for SRH information and services is highest among the most vulnerable—adolescents, populations with low socioeconomic status, those living in rural areas and urban slums, people living with HIV, internally displaced people and those living in humanitarian crises contexts.^1^ We define humanitarian crises as a significant disruption of the functioning of a community or society causing widespread human, material, economic or environmental losses, which exceed the ability of those affected to cope using its own resources, necessitating a request to the national or international level for external assistance. The crisis situation may be man-made (eg, armed conflict) or natural (eg, drought). Individuals living in humanitarian crises face significant barriers and challenges, which impede their access to healthcare more generally and sexual and reproductive healthcare in particular.^1^ These challenges are particularly exacerbated for women and girls.

Women and girls are affected significantly in both sudden and slow-onset humanitarian crises, and face multiple SRH challenges in these contexts—there are an estimated 32 million women and girls of reproductive age (ie, 15–49 years) living in humanitarian crises situations, all of whom need SRH information and services.^2^ Humanitarian crises can increase the risk of poor SRH outcomes due to reduced access to services and supplies, damaged health facilities and increased exposure to sexual violence among other factors, and have implications across the life cycle, eg, increased risk of maternal and newborn morbidity and mortality.

In order to address this need, the Inter-Agency Working Group on Reproductive Health in Crisis (IAWG), a consortium of non-governmental organisations, donors, governments and the United Nations agencies, developed a technical and programmatic guide, *Reproductive Health in Refugee Situations: An Inter-Agency Field Manual*, to provide guidance to field staff on reproductive health interventions during emergencies.^3^ Specifically, the *Field Manual* includes a chapter on the Minimum Initial Service Package (MISP), to be implemented at the onset of every humanitarian emergency, via the following four programme pillars: (i) prevent and manage the consequences of sexual violence; (ii) reduce HIV transmission; (iii) prevent maternal and newborn death and illness and (iv) plan for comprehensive sexual and reproductive health care, integrated into primary healthcare, as the humanitarian crises situation permits.

While some progress has been made with regard to the availability of SRH services in humanitarian crises (eg, funding for certain services), significant challenges remain with regard to utilisation of these services. These challenges are further compounded by overall gaps in health systems capacity. Recent systematic reviews have found that the absence of quality data on women’s, children’s and adolescents’ health in emergencies hinders design and implementation of sustainable interventions.^1 4^ Another systematic review by Warren *et al.* highlighted evidence gaps in the effectiveness of delivering and scaling-up public health interventions in humanitarian crises, as well as in evidence for populations using SRH services.^5^ It also indicated that the MISP was not being systematically implemented, and that core SRH services were being neglected (eg abortion, contraception, care for adolescents). Failure to implement the essential SRH services as an integrated package has significant consequences for individuals in humanitarian crises settings. Furthermore, the population of displaced individuals has increased in recent decades, and increased resources are needed to improve health outcomes.^4^ A recent systematic review noted the dearth of studies measuring SRH outcomes in humanitarian crises^4^; however, a key challenge in improving utilisation of quality SRH services has also been a lack of evidence, and there has been no systematic review to date that has assessed the utilisation of these services in crises settings. Data are needed to inform how to most effectively increase utilisation of services, and to advocate for the SRH needs of individuals living in humanitarian crises.

To build on progress made, we aimed to consolidate existing evidence for the utilisation of SRH interventions, including the MISP, from the onset of emergencies by conducting a systematic review. This study aimed to assess the utilisation of SRH services in humanitarian crises in low- and middle-income countries from 1980 to 2017. We also aimed to assess issues related to access of SRH interventions including the MISP, eg, whether these services are being used by vulnerable populations such as people with disabilities, sex workers, adolescent girls and lesbian, gay, bisexual, transsexual, queer and intersex populations.

## Methods

This systematic literature review adheres to the Preferred Reporting Items for Systematic Reviews and Meta-Analyses statement.^6^ It is registered with the PROSPERO database with identifier number CRD42017082191.

### Search strategy and selection criteria

Search terms for SRH were based on the standardised definition from the International Conference on Population and Development in 1994 and the WHO Reproductive Health Strategy,^7 8^ as well as a previous systematic review by Warren *et al.* on SRH interventions in humanitarian crises settings.^5^ Our search encompassed terms used in international guidelines on reproductive health in conflict-affected situations, including family planning, abortion, HIV/AIDS and sexually transmitted infections (STIs), prevention of mother-to-child transmission (PMTCT), maternal and newborn health and sexual and gender-based violence. Although humanitarian crises can affect any country, only studies from low-income or middle-income countries were included in this study, as the majority of humanitarian crises occur in these countries, and the resources available to address them are different in high-income countries.

We searched literature from 1 January 1980 to 10 April 2017 as a recent literature review on public health interventions in humanitarian crises yielded no SRH studies published prior to 1980.^4^ A detailed protocol with specific search terms and strategy is provided in online supplementary appendix A. This was generated by the authors and expert review, and supplemented by searching for other search strategies used in previous systematic reviews on similar topics.^1 4 5^ We also consulted a trained information science and Cochrane review specialist to review our literature searching syntax and strategy. The search strategy followed the following formula: (humanitarian crises-related terms) and (SRH domain, eg contraception-related terms) and (utilisation-related search terms) and (study type-related terms) and (year range) and (low- and middle-income country-related terms).

10.1136/bmjgh-2017-000682.supp1 Supplementary file 1

We included quantitative and qualitative studies from peer-reviewed journals across the following four databases: EMBASE, Global Health, MEDLINE and PsychINFO. We complemented searches by screening the reference lists of (1) papers for potentially relevant studies and (2) relevant systematic reviews. We also consulted experts on SRH service delivery and research to identify research not found during our systematic search.

### Data analysis

**Figure 1:**
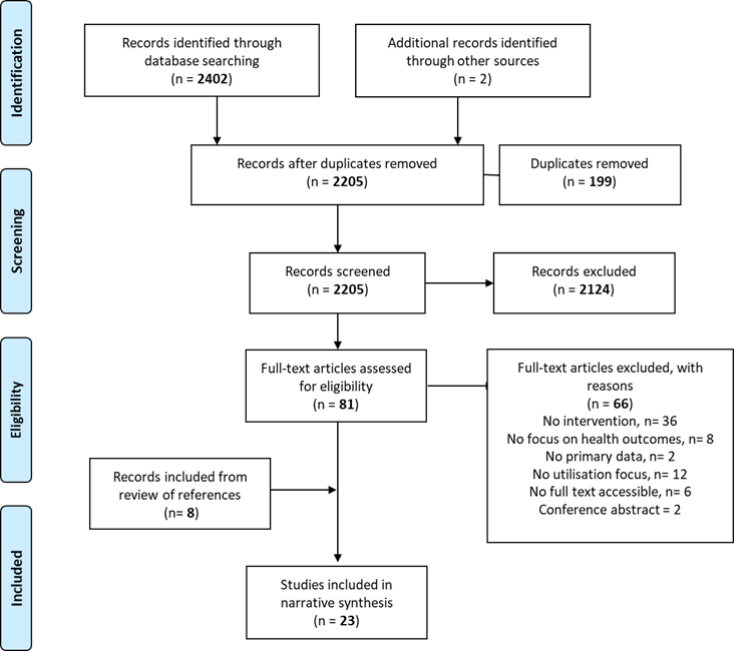
Preferred Reporting Items for Systematic Reviews and Meta-Analyses (PRISMA) flow chart: selection process for systematic review on the utilisation of sexual and reproductive health interventions in humanitarian crises settings.

We downloaded all returned citations from the searched databases into an Endnote library and applied a standard data-screening process (figure 1). Primary and secondary outcomes and primary outputs of interest for inclusion were based on those from the IAWG field manual.^3^ This is an established and widely used manual for SRH in crisis settings, and was selected based on discussion with an SRH expert committee. Inclusion and exclusion criteria applied during the screening process are outlined in table 1. Data from the final selected studies were extracted into an Excel database, with data extraction fields including study author and year, setting, target population, crisis type, crisis stage, study design and methods, research setting, study outcomes and intervention descriptions. Two researchers independently conducted screening of titles and abstracts, followed by data extraction from full-text review. Discrepancies in data extraction were resolved by arbitration by an additional independent researcher.

**Table 1:** Inclusion and exclusion criteria

Category	Included	Excluded
Population of interest	Crisis-affected populations receiving humanitarian assistance or aid in low-income or middle-income countries (as defined by World Bank, 2012): including refugees and internally displaced persons (see online supplementary appendix A for definitions of these terms)	
Intervention	Any health-related intervention seeking to improve sexual and reproductive health (SRH) outcomes	
Outcomes of interest	*Primary quantitative research studies:* indicators included self-reported use and/or confirmed use of the Minimum Initial Service Package (MISP) services (ie, use of emergency obstetric and newborn care services, use of condoms and/or contraceptives, use of HIV and sexually transmitted infection screening services, use of abortion services, use of adolescent health services and use of psychological support services by sexual violence survivors). Self-reported use is defined as when a beneficiary reports the use of a product or service without additional verification; this indicator is often biased. Confirmed use is defined as when the evaluation tests, observes or confirms a product or service was used in some way (e.g. via observation). *Primary qualitative research studies:* we included outcomes exploring the determinants of service utilisation of the MISP and its components.	Studies which do not evaluate utilisation of SRH interventions
Study types and design	*Primary quantitative research studies:* Study designs including randomised controlled trials, non-randomised controlled trials, controlled before-after studies, controlled interrupted time series studies, economic studies (cost-effectiveness analysis, cost-utility analysis, cost-benefit analysis, economic modelling) of public health which the outcome is measured before and after the intervention or an intervention is studied against another intervention with baseline or control group. *Primary qualitative research studies:* Study designs including focus group discussions, in-depth interviews and structured observations, limiting studies to those with an observable contrast of time and/or a control group.	Studies on preparedness or resilience if not linked to an intervention evaluating the utilisation of SRH interventions Studies with no specific health intervention and no outcomes (ie, studies examining only health needs, prevalence, health risk-factors, co-ordination). Review papers; references listed in review papers were screened to find more primary data sources
Data type	Must include primary data	Includes only secondary data
Phase of humanitarian crises	Studies conducted during the acute, chronic and early recovery phases of a humanitarian crisis (defined in online supplementary appendix A).	Studies conducted after a humanitarian crisis has stabilised
Publication date	1 January 1980–10 April 2017	
Language	English, French	Other languages

We used a narrative synthesis approach due to the heterogeneity of study outcomes, interventions and methods.^9^ The narrative synthesis approach comprises researchers moving in an iterative manner between the following four elements: (i) developing a theory of how the intervention works, why and for whom; (ii) developing a preliminary synthesis of findings of included studies; (iii) exploring relationships in the data and (iv) assessing the robustness of the synthesis. Findings were synthesised by main SRH outcomes, ie, family planning; abortion; prevention, treatment, care for STIs including HIV/AIDS; maternal and newborn health, including obstetric care; gender-based violence and studies with cross-cutting SRH themes. These themes were developed iteratively after conducting thematic analysis of the stated aims and primary reported health outcome of interest of the studies, which was done by manually coding the papers and reviewing data extracted to the Excel database.

The quality of reporting in the included studies was assessed using the Strengthening the Reporting of Observational Studies in Epidemiology (STROBE) and Critical Appraisal Skills Programme (CASP) checklists,^10 11^ which are commonly used for reporting quality of observational and qualitative studies, respectively. The STROBE checklist includes measures on reporting of participant selection, variables, data sources, bias, descriptive and outcome data, interpretation and generalisability, among others. The CASP checklist includes measures on reporting of clear aims, use of appropriate methodology, research design and participant selection, adequate consideration of the relationship between the researcher and participants, rigour of data analysis, among others. To further explore the quality of reporting, we awarded papers one point for reporting each of the items on the relevant checklist. When totalled, these points formed the numerator of a proportional score, with the denominator being the total number of possible relevant checklist items. This denominator varied slightly by study type, that is, 22 points for the STROBE checklist and 10 points for the CASP checklist. NSS, SA and JS conducted quality assessment; each independently evaluated the quality of all included studies and discussed each discrepancy until consensus was reached. The study team determined a priori that papers were considering low reporting quality if the scoring was <33%, moderate quality if 34%–66% and high quality if >67%. These quality thresholds have been used in a previous systematic review on SRH in humanitarian crises settings.^5^

### Role of the funding source

The funder of the study had no role in study design, data collection, data analysis or data interpretation. The corresponding author had full access to all data in the study and had final responsibility for the decision to submit for publication.

### Ethics

Ethics approval was not applicable.

## Results

We screened titles and abstracts of 2404 citations, 2402 from literature searches and 2 from screening reference lists of other relevant systematic reviews. After full-text screening, 23 studies met our inclusion criteria and were selected for final review (figure 1). We identified studies focusing on family planning, HIV/STIs, maternal and newborn health and gender-based violence. We found no studies with interventions specifically focusing on PMTCT of HIV, vaginal injuries and fistula, post-abortion care, safe abortion, prevention of sexual violence and comprehensive clinical management of rape.

The 23 included studies were conducted in 16 countries with populations affected by armed conflict, natural disaster or both (table 2). Between 1997 and 2009, nine papers were published that met our inclusion criteria. Between 2010 and 2015, 14 more papers were found. Of these 23 studies, 22 used cross-sectional, pre-post and cohort study designs. One paper included in this review used qualitative research methods, specifically focus group discussions.

**Table 2:** Study characteristics

Lead author and year	Country	Setting	Population	Crisis type	Crisis stage	Study design	Intervention	Quality
Curry *et al*^16^ 2015	Chad, DRC, Djibouti, Mali, Pakistan	Rural/urban	General/refugee/IDP	Armed conflict and natural disaster	Acute	Cross-sectional	Training, facility supervision, supply of contraceptives, community mobilisation and awareness raising	Moderate
Howard *et al*^25^ 2008	Guinea	Camp	Refugee	Armed conflict	Stabilised	Cross-sectional	Development of a refugee-led ‘Reproductive Health Group’	High
Huber *et al*^17^ 2010	Afghanistan	Rural	General	Armed conflict	Chronic	Prepost study	Health education, community health workers delivery of injectable contraceptives	Moderate
Casey *et al*^26^ 2013	Uganda	Rural	IDP/general	Armed conflict	Stabilised	Cross-sectional	Mobile outreach and public health centre strengthening	High
Adam^27^ 2016	Sudan	Camp	IDP	Armed conflict	Chronic	Cross-sectional	Home counselling and awareness raising	High
Raheel *et al*^28^ 2012	Pakistan	Urban	Refugee	Armed conflict	Stabilised	Cross-sectional	Subsidised healthcare (90% subsidies for doctor’s visits, hospital visits, emergency care, free family planning, excluding prescriptions)	High
Casey *et al*^29^ 2006	Sierra Leone	Urban	General/IDP	Armed conflict	Chronic (stabilised)	Prepost study	HIV prevention activities	High
Logie *et al*^30^ 2014	Haiti	Camp	IDP	Natural disaster	Chronic (stabilised)	Cohort	Psycho-educational HIV/STI prevention delivered by peer health workers	High
Culbert *et al*^18^ 2007	DRC	Urban	General	Armed conflict	Chronic (acute)	Cohort	Initiation of antiretroviral treatment	Moderate
Goodrich *et al*^19^ 2013	Kenya	Rural/camp	General/IDP	Armed conflict	Acute	Prepost study	Rapid information dissemination, disaster response plan (including outreach activities)	Moderate
Adam^31^ 2015	Sudan	Camp	IDP	Armed conflict	Chronic (acute)	Cross-sectional	Interpersonal communication and mass education campaigns	High
Groppi *et al*^20^ 2015	South Sudan	Urban/rural	General	Armed conflict	Chronic (acute)	Cross-sectional	Ambulance-based referral system to hospital for emergency obstetric care	Moderate
Hadi *et al*^32^ 2007	Afghanistan	Rural	General	Armed conflict	Chronic (stabilised)	Prepost study	Introduction of a community-based safe motherhood programme	High
Fujiya *et al*^21^ 2007	Occupied Palestinian Territories	Urban	General	Armed conflict	Acute	Prepost study	Health insurance scheme and reduction in cost of hospital-based deliveries	Moderate
Kandeh *et al*^12^ 1997	Sierra Leone	Rural	General	Armed conflict	Acute	Prepost study	Distribution of drug kits, staff training, facility renovation, training and deployment of community motivators	Low
Ayoya *et al*^15^ 2013	Haiti	Urban	General	Natural disaster	Acute	Cross-sectional	Baby tent breastfeeding stations	Low
Purdin *et al*^22^ 2009	Pakistan	Urban/rural	Refugee	Armed conflict	Chronic (acute)	Prepost study	Comprehensive emergency obstetric care programme	Moderate
Leigh *et al*^23^ 1997	Sierra Leone	Rural	General	Armed conflict	Chronic (acute)	Prepost study	Staff recruitment, staff training, operating theatre and blood bank setup	Moderate
Samai and Sengeh^24^ 1997	Sierra Leone	Urban	General	Armed conflict	Acute	Controlled pre-post	Four-wheel drive vehicle posted at hospital for referrals, with motorbikes posted to facilitate connections with rural areas	Moderate
Tanabe *et al*^34^ 2013	Myanmar	Urban/rural	General	Armed conflict	Chronic (acute)	Qualitative	Community-based medical care for survivors of sexual assault, with referrals to mobile clinics	High
Qayum *et al*^13^ 2013	Pakistan	Camp	IDP	Armed conflict	Chronic (stabilised)	Cross-sectional	MISP with focus on clean delivery kits	Low
von Roenne *et al*^14^ 2010	Guinea	Rural	Refugee	Armed conflict	Chronic (stabilised)	Cross-sectional	Training of lay reproductive health facilitators and staff, service provision from medically trained refugees	Low
Mullany *et al*^33^ 2010	Myanmar	Rural	IDP	Armed conflict	Chronic (stabilised)	Cross-sectional	Three-tiered network of community-based providers: (1) traditional birth attendants; (2) health workers; (3) maternal health workers	High

### Study quality

Of the 23 studies, 22 (95.7%) were assessed for quality of reporting using the STROBE checklist and the only qualitative study (4.3%) was assessed using the CASP checklist for qualitative studies. Of the observational studies, four (17.4%) were found to be of low quality,^12–15^ nine (39.1%) were of moderate quality^16–24^ and the remaining nine (39.1%) were of high quality.^25–33^ The included qualitative study was also deemed to be high quality.^34^

Overall, there were common areas in which the included studies in this review provided low quality reporting. First, all the moderate quality and low quality quantitative studies stated changes in utilisation outcomes, but statistical associations between the intervention and the outcome were not given. Second, it was also not clear whether relevant confounders and biases were considered during the design of the study and analysis of the data.

The following section synthesises findings on the utilisation of SRH interventions in humanitarian crises settings by main SRH outcomes.

### Family planning

We identified six studies whose primary aim was to evaluate an intervention to improve the use of family planning services in humanitarian crises settings. A pre-post, multi-site study of moderate quality conducted in Chad, Democratic Republic of Congo (DRC), Mali, Djibouti and Pakistan evaluated the impact of staff training, facility supervision, supply of contraceptives as well as community awareness and mobilisation activities on the contraceptive uptake among women of reproductive age. The study found a general increase in new users of modern family planning methods over time, notably for new users choosing long-acting and reversible contraceptives (78% in the DRC, 72% in Chad, 51% in Mali, 29% in Pakistan).^16^ A study of high quality assessing a home-based counselling and awareness programme for internally displaced women in Sudan led to an increase in the use of modern family planning methods (adjusted OR (aOR) 2.8, 95% CI 2.0 to 4.1).^27^ Another study of high quality assessed a refugee-led reproductive health group operating across 48 Guinean refugee camps.^25^ The intervention recruited refugee nurses and midwives to local health facilities, trained lay women to provide health education and contraception and to facilitate referrals. Individuals who reported the reproductive health group facilitators as their primary source of information increased their use of contraception (aOR 1.3, 95% CI 0.7 to 4.2, non-significant). In Uganda, a high quality study assessing a mobile outreach intervention and public health strengthening programme led to an increase in the number of women who reported ever using a family planning method (aOR 2.23, 95% CI 1.7 to 2.92, P<0.001), and a reduction in the unmet need for family planning from 52.1% to 35.7% (aOR 0.47, 95% CI 0.37 to 0.6, P<0.001).^26^ A study of moderate quality in rural Afghanistan assessed the impact of health education and the delivery of injectable contraceptives by community health workers (CHWs).^17^ Over an 8-month period in 2005–2006, contraceptive use increased by 24%–27% across three sites.^17^ Another high quality study in Pakistan assessed the provision of subsidised healthcare to refugees, reporting use of contraceptives in the subsidised group (54%) was more than double the use reported in the non-subsidised group (25%) (P<0.001); and reporting that the non-subsidised group was more likely to use the pill (40.7%), whereas the subsidised group was more likely to have tubal ligation (36.7%) (P<0.001).^28^

### Prevention, treatment and care for HIV/AIDS and STIs

We identified four studies that focused on improving the utilisation of prevention, treatment and care services for HIV/AIDS and STIs. Two high quality studies evaluated the impact of HIV prevention interventions on condom use. Casey *et al.* assessed HIV prevention education activities and their subsequent effect on contraceptive use among adolescents aged 15–24 years using a pre-post study design.^29^ The study found that at baseline, approximately 15.6% of female and male adolescents reported using a condom the last time they had sex, while this proportion increased to 46.2% (P<0.01) and 37.1% (P<0.01), respectively after the intervention. Additionally, 24.8% of adolescents reported ever having used a condom at baseline, while this increased to 63.6% post-intervention.^29^ A cohort study conducted in Haiti after the 2012 earthquake aimed to evaluate a psycho-educational HIV/STI prevention programme that was delivered by female peer health workers.^30^ The programme consisted of an HIV/STI educational video-based session, and a 6-week psycho-educational programme with weekly group meetings. A statistically significant change in condom use was identified post-intervention, after adjusting for confounders (OR 4.05, 95% CI 1.86 to 8.83, P<0.05).^30^

Two moderate quality studies specifically focused on assessing the utilisation of antiretroviral therapy (ART). The first, a pre-post study conducted in a camp setting during postelection violence in Kenya, evaluated a disaster response plan that included rapid information dissemination through the media, HIV outreach activities and a medical record system helping to track missed appointments among HIV-positive patients.^19^ Using clinic attendance as the primary outcome, the study reported that the majority of clinics (60.0%, n=12) had more visits during their opening week, and that there was an overall increase in patient volume by 105% compared with the baseline. The number of unscheduled visits also increased from 27.2% pre-intervention to 42.4% three months after clinics were opened. Additionally, there was a decline in the number of missed patient visits, although an exact proportion of decrease was not reported. The second was a cohort study conducted in the DRC to evaluate an ART intervention that included establishing voluntary counselling and testing sites in addition to HIV clinics.^18^ During the four years of the programme, 110 076 people received voluntary counselling and testing, and of these people, 19% tested HIV positive. Ninety-four per cent of those testing positive received care in HIV clinics. By the fourth year of the programme, 26% of patients had commenced ART. Good ART adherence was achieved by 99% of patients.

### Maternal and newborn health including obstetric care

Ten studies were identified whose primary aim was to improve maternal, obstetric and newborn health services. Of these, three studies specifically focused on increasing service utilisation of maternal healthcare in humanitarian crises settings.^20 31 32^ Adam conducted a high quality cross-sectional study to evaluate an intervention aiming to increase antenatal care (ANC) attendance among recently pregnant women aged 15–48 years using interpersonal communication and mass education campaigns over a 2-year time period.^31^ These education campaigns were found to increase the likelihood of a pregnant woman attending at least three ANC visits (OR 8.8, 95% CI 6.4 to 12), and having at least three tetanus doses (OR 2.5, 95% CI 1.9 to 3.3). There were also increases in the likelihood of the woman having a facility-based delivery (OR 5.4, 95% CI 4.0 to 7.4, P<0.001) and attending at least one postnatal care visit (OR 5.5, 95% CI 4.0 to 7.7, P<0.001). In a high quality study, Hadi *et al.* examined the effect of introducing a community-based safe motherhood programme in Afghanistan on utilisation of ANC services and institutional delivery.^32^ In both instances, there was statistical evidence of an increase in service utilisation compared to baseline. There was a 53.9% increase (P<0.01) in ANC services received from 2004 to 2006, and a 23.9% increase (P<0.01) in facility-based deliveries over the 2-year time period of the study.^32^

Fujiya *et al.* used a moderate quality pre-post study to evaluate the effect of a health insurance scheme and a reduction in the cost of hospital-based deliveries in the Occupied Palestinian Territories during an Israeli military invasion.^21^ There was a 9.1% increase in live births at government hospitals when health insurance was provided to pregnant women. There was also a consistent increase in the number of births at the study hospital once the price was reduced for facility-based deliveries.

Additionally, we identified two studies that reviewed interventions aiming to reduce the unmet needs for emergency obstetric care (EmOC). In a low quality pre-post study, Kandeh *et al.* evaluated the impact of an intervention in Sierra Leone between 1992 and 1995 that included the distribution of drug commodities to health units, staff training, facility renovation and the use of community motivators.^12^ An analysis of met needs during implementation showed that health facilities were seeing 43% of the total expected number of women with complications in the area during the first study period, prior to most interventions, with this figure rising to 59% after full implementation of the intervention. A moderate quality pre-post study conducted in Pakistan also evaluated the effect of a comprehensive EmOC programme on facility-based deliveries.^23^ During the intervention period, the proportion of births in an EmOC facility increased from 4.8% at baseline to 67.2% post-intervention.

One pre-post study of moderate quality was conducted in Sierra Leone between 1990 and 1995 to assess an intervention with the specific aim of increasing facility utilisation by improving the infrastructure of a rural health facility, and by providing training.^12^ Additional staff were recruited and trained in obstetric care, and an operating theatre and blood bank were established. The study found that maternal admissions more than doubled in the first year of the intervention when only the first medical officer with obstetric skills was posted. There was a fourfold increase of maternal admissions of those with obstetric complications.^12^

Two moderate quality studies were identified that evaluated vehicle-based interventions to increase the use of obstetric services in emergencies. Samai *et al.* used a controlled pre-post study to evaluate the impact of a four-wheel vehicle that was posted to a government hospital in Sierra Leone, with motorbikes to provide additional linkages between primary health units and the vehicle.^24^ The intervention also included community education activities, provision of drugs and improvement to the infrastructure of the hospital. A radio communication referral system was also established to improve communication between the hospital and rural areas. The study found that utilisation of EmOC services doubled, but that this improvement could also be attributed to improved community awareness of obstetric emergencies and improved care at the hospital.^24^ Groppi *et al.* used a cross-sectional study to determine the impact of an ambulance-based referral system to a hospital for EmOC services in South Sudan by collecting data on facility deliveries prospectively.^20^ One year post-intervention, 13.3% of the expected deliveries in the hospital’s catchment area took place at the facility. The study found that 22.0% of all ambulance referrals to the hospital that year were for women with major obstetric complications.

Lastly, one low quality study assessed an intervention aiming to improve the use of neonatal and child care services by assessing the utilisation of baby tent breastfeeding stations in Haiti by breastfeeding women after a natural disaster, using a cross-sectional study design.^15^ The study reported that 54% of the infants enrolled at a breastfeeding station were aged <6 months, and that 70% of these infants were exclusively breastfed. The remaining infants were reported to be receiving ‘mixed feeding’, but 10% moved to exclusive breast feeding by the end of their participation in the programme.^15^

### Gender-based violence

One high quality qualitative study was identified whose primary aim was to provide data on the feasibility of providing community-based medical care for sexual assault survivors as part of a larger process evaluation of the intervention.^34^ Conducted in Myanmar in an armed conflict setting, this study used focus group discussions with community members, traditional birth attendants and CHWs to understand the utilisation of a community-based medical care package for survivors of sexual assault delivered at the community level. This package was adapted from the 2004 WHO’s Clinical Management of Rape Survivors facility-based protocol so that it could be delivered by CHWs. The study found that some of the perceived barriers and challenges for sexual assault survivors in accessing and using services are shyness, shame, fear of others’ opinions and fear that they may be denied help. It was also suggested that generally, the community needed to feel more comfortable in seeking care from CHWs.^34^

### Interventions targeting outcomes across multiple SRH domains

In total, three studies were identified that evaluated interventions that focused on more than one of the SRH domains mentioned above. One low quality study used a household cross-sectional survey to evaluate whether internally displaced people within a camp setting in Pakistan were able to access the MISP, and implemented services with a special focus on the provision of clean delivery kits.^13^ Findings report 23% of women had problems in accessing ANC and delivery services at the health facilities, and 87% of women answered that they did not receive follow-up visits or check-ups during their pregnancy by a health worker. Over three-quarters (78%) of the women surveyed also reported they had not received clean delivery kits.^13^

The remaining two articles were both cross-sectional studies and evaluated interventions providing family planning and maternal health services. One low quality study evaluated an intervention in which lay reproductive health facilitators and staff recruited from the refugee population in Guinea were trained in the provision of family planning and maternal health services.^14^ The study found that the contraceptive prevalence rate among refugees in the intervention’s catchment area was 25% higher than the national country average, and that ANC coverage was 54% in its catchment area post-intervention, which was higher than the estimated ANC coverage average (ranging from 11% to 42%) among the refugee population in Guinea prior to implementation. Lastly, a high quality study evaluated the impact of a three-tiered network of community-based providers in Myanmar, which included traditional birth attendants, health workers and maternal health workers.^33^ With this intervention, the use of modern contraception methods increased from 23.9% to 45.0% (prevalence rate ratio (PRR)=1.9, 95% CI 1.6 to 2.2). The number of women receiving at least one ANC visit or more increased from 39.3% to 71.8% (PRR=1.8, 95% CI 1.6 to 2.0). Deliveries were also 9.6 times more likely to be attended by a provider trained in EmOC post-intervention (PRR=9.6, 95% CI 7.2 to 12.6).^33^

## Discussion

This is the first systematic review to assess the utilisation of SRH interventions, including the MISP, in humanitarian crises. Of the 2404 citations screened, the review identified 23 studies since 1980 assessing the utilisation of SRH services in humanitarian crises that met the inclusion criteria. Interventions resulting in increased SRH service utilisation included interpersonal and peer-led education and mass media campaigns, focused community-based programming and tiered community health service provision to improve reproductive and maternal health services. Lower quality evidence was found to support general health system strengthening efforts combined with the use of community motivators, birth preparedness interventions and the training of lay SRH facilitators to provide family planning and ANC. We identified no studies that evaluated the utilisation of interventions focused on PMTCT, STI treatment and prevention excluding HIV/AIDS, vaginal injuries and fistula, post-abortion care, safe abortion, prevention of sexual violence, comprehensive clinical management of rape or of the MISP as a comprehensive package of interventions.

The quality of evidence identified in this review was variable, with particularly weak study design and most studies graded as high and moderate quality, since study design was only one component in the STROBE and CASP checklists used to assess study quality. Just over one-third (36.7%) of the studies (n=7) used quasi-experimental study designs, which provided some statistical measure of difference between intervention and outcome. This also meant that, where appropriate, there was insufficient adjustment for potential confounders. Evidence on attribution is particularly weak, with the vast majority of studies using a cross-sectional or pre-post study design, with no control group. Qualitative studies have the potential to contribute rich perspectives from study populations on service utilisation, but we found only one study using this design, and no study using mixed methods to assess SRH service utilisation in humanitarian crises. Overall, only 39.1% of the studies (n=9) selected were graded as high quality, 39.1% as moderate quality (n=9) and 17.4% as low quality (n=4). There was limited use of stratification, for example, by gender or age, and so we were not able to capture potentially differing health utilisation outcomes in more vulnerable group such as adolescent girls. Use of STROBE and CASP tools identified fundamental methodological issues with most of the available evidence including: lack of appropriate study design; absence of reporting on sampling methods; lack of control groups and randomisation procedures; limited appreciation of clear exposures and confounders and an inadequate handling of bias. Statistical analysis was reported in only five studies (21.7%), signalling lack of robust research in this field. A recent systematic review on public health interventions in humanitarian crises found a greater share of studies on communicable diseases and nutrition in humanitarian crises,^4^ suggesting that more rigorous SRH research is possible in these settings. We did not identify any cost-effectiveness studies, which is of concern as it highlights gaps in the economic data required by policymakers and programme implementers to deliver and scale up SRH services.

We found a similar level of evidence and quality of studies measuring SRH utilisation compared with previous systematic reviews evaluating SRH interventions in humanitarian crises,^1 5^ signalling slower progress in advancing the evidence base in this field. It was not possible to synthesise findings by setting (e.g. camps, urban areas) or population type (e.g. adolescents), given the paucity of identified studies. Casey *et al.*^29^ found that adolescents access health services in different ways than the general population and have different concerns (e.g. privacy) and health needs, signalling the importance of conducting studies that specifically target this population and assess their utilisation of SRH services.^29^ In addition to research on delivering and scaling up SRH services to different target populations, we should also consider implementation research in different humanitarian crises settings. For example, the private sector and informal providers may play a larger role in service delivery in protracted humanitarian crises than in acute settings where non-governmental organisations may be delivering most of the services. These evidence gaps point to the need for robust and timely research on the mechanisms through which SRH interventions can increase utilisation by key populations across a variety of humanitarian crises.

This review has a number of limitations. Although we included qualitative and quantitative studies in our study design criteria, other narrow inclusion criteria may have led to the exclusion of some peer-reviewed literature. Additionally, our language inclusion criteria, i.e. only studies published in English or French, imposed by the capacity of the study team, may have limited the numbers of citations returned by our search. We also note that the STROBE checklist only assesses reporting of studies, rather than the actual design, sampling/recruitment and the appropriateness of analysis methods. Study outcomes, intervention types and methods varied widely across the 23 included studies, which did not allow us to conduct a meta-analysis. We also acknowledge that the varying geography, social and cultural diversity of study settings and populations included in our review would have an impact on the utilisation of SRH interventions in these settings, and might lead to limited external validity for some SRH interventions. However, no studies reported measuring these distal determinants, so we were unable to assess their effect on the utilisation of SRH interventions.

## Conclusions

The review found some evidence to support increased utilisation of SRH services through peer-led and interpersonal education and mass media campaigns, community-based programming and a three-tiered network of community-based providers for reproductive and maternal health services. However, these results should be interpreted cautiously due to limited use of statistical data and less robust study design. Rigorous and timely research, as well as high quality evidence to support the use of different intervention designs, is needed to identify evidence-based interventions to increase the utilisation of SRH services by key populations across a range of diverse humanitarian crises settings.
