# Supplementary material for: A long way to go: a systematic review to assess the utilisation of sexual and reproductive health services during humanitarian crises
Source: BMJ Glob Health. 2018 May 3;3(2):e000682. doi: 10.1136/bmjgh-2017-000682 (PMC5935157; doi:10.1136/bmjgh-2017-000682)
Supplement: Supplementary file 1 [file bmjgh-2017-000682supp001.pdf]

Appendix A - Systematic review protocol

Systematic Literature Review on the utilisation of Sexual and Reproductive Health (SRH) interventions in Humanitarian Crises Settings

1. Aim

We aim to conduct a mixed-methods systematic review to address the following question: *what is the evidence base for the service utilisation of SRH interventions including the MISP from the onset of emergencies?*

Specifically, we will focus on examining the evidence base for the change in service-use over time of the MISP overall and its four pillars (i.e. Use of EmONC services, use of condoms and/or contraceptives, use of HIV and STI screening services, use of abortion services, use of adolescent health services, and use of psychological support services by sexual violence survivors).

Determinants of service utilisation of SRH interventions including the MISP will also be examined to inform the quantified change in service-use (i.e. Population targeting, costs, knowledge of services, perceived quality of services through provider/client satisfaction).

2. Methods

This systematic literature review aims to provide a situational analysis of the existing evidence on the service utilisation of SRH interventions including the Minimum Initial Service Package (MISP) in humanitarian crises settings from January 1, 1980 to April 10, 2017.

As this review will use both quantitative and qualitative data, the systematic review methodology will adhere to both the Preferred Reporting Items for Systematic Reviews and Meta-Analyses (PRISMA) statement for quantitative data [2] and the Critical Appraisal Skills Programme (CASP) tool for Qualitative Research. [5]

2.1 Key terms:

The following key terms and concepts relate to this systematic literature review, their definitions having been adapted from the World Health Organization (WHO) Humanitarian Health Action Dictionary. [3]

<u>Public Health Intervention</u>: Public health actions that seek to improve health outcomes.

<u>Humanitarian crisis</u>: A serious disruption of the functioning of a community or a society causing widespread human, material, economic or environmental losses which exceed the ability of the affected community or society to cope using its own resources, necessitating a request to national or international level for external assistance. The disaster situation may be either manmade (e.g. armed conflict) or a natural phenomenon (e.g. drought).

<u>Man-made humanitarian disasters</u>: These include international armed conflicts; non-international armed conflicts; and other situations of violence. [4]

Natural disasters: These include hazardous natural phenomena leading to humanitarian crises such as earthquakes; volcanic activity; landslides; tsunamis; tropical cyclones and other severe storms; tornadoes and high winds; floods, and droughts.

Early Recovery: Early Recovery is defined as recovery that begins early in a humanitarian setting. It is a multi-dimensional process, guided by development principles. It aims to generate self-sustaining nationally owned and resilient processes for post-crisis recovery.

2.2 Search strategy and search terms

This literature review will use peer-reviewed literature, which will be located using the following electronic bibliographic databases: Medline, Embase, Global Health, and PsychINFO. The search structure will consist of the following:

- terms related to humanitarian crises;
- AND terms related to public health interventions;
- AND terms related to lower and middle income economies;
- AND terms related to SRH interventions including the MISP
- AND terms related to service utilisation

The search terms used for the main bibliographic databases are given in Section 2. The additional specific search terms related to the MISP and service utilisation will then be added on (see Section 3 and 4).

Searches were supplemented by reviewing the reference lists ('references of references') of selected articles to find any other relevant papers.

2.3 Inclusion/exclusion criteria

The following seven key inclusion criteria will be used in this review:

- Types of studies: *Primary quantitative research studies.* Study designs including randomised controlled trials, non-randomised controlled trials, controlled before-after studies, controlled interrupted time series studies, economic studies (cost-effectiveness analysis, cost-utility analysis, cost-benefit analysis, economic modelling) of public health which the outcome is measured before and after the intervention or an intervention is studied against another intervention with baseline or control group.
  Primary qualitative studies. Study designs including focus group discussions, in-depth interviews, and structured observations, limiting studies to those with an observable contrast of time and/or a control group.

- Populations of interest: Populations affected by humanitarian crises and receiving humanitarian assistance in low and middle-income countries (based upon World Bank country classification).

- Health outputs of interest: *Primary quantitative research studies:* To assess service utilisation, specific indicators used will include self-reported use and/or confirmed use of MISP services (i.e. use of EmONC services, use of condoms and/or contraceptives, use of HIV and STI screening services, use of abortion services, use of adolescent health services, and use of psychological support services by sexual violence survivors). Self-reported use is when a beneficiary reports the use of a product or service without additional verification; this indicator is often biased.

Confirmed use is when the evaluation tests, observes or confirms a product or service was used in some way (e.g. via observation).
Primary qualitative research studies: Studies that use focus group discussions, in-depth interviews, or semi-structured interviews to explore the determinants of service utilisation of the MISP and its components will be included.

- Crisis Phase: Studies that occur in humanitarian crises including those that evaluate: i) the impact of preparedness and resilience on public health outcomes during a humanitarian crises and/or ii) studies that evaluate the impact of public health interventions during the acute, chronic, or early recovery phases of humanitarian crises. An acute phase is defined as an outbreak of a crisis situation which results in destruction of lives and property, as well as population displacements. Chronic phase encompasses a prolonged crisis, when displaced populations (e.g. forced migrants) are settled in temporary locations, either in camps or within host communities. Early recovery phase is defined as when the affected population(s) are in a more stable period of transition.

- Data type(s): Must include primary data.

- Date of intervention and publication: January 1, 1980 – April 10, 2017.

- Publication language: English, French.

The following criteria will be used to exclude studies from this review:

- Studies with no specific health intervention and no outputs (i.e., excluding studies that examine only health needs, prevalence, health risk-factors, health outcomes, co-ordination).
- Studies that examine preparedness and resilience not linked to health outcomes in humanitarian crises (e.g. studies on housing fortification before flooding).
- Review papers; only references listed in review papers were screened to find more primary data sources.

2.4 Study screening and data extraction

The systematic literature review will be conducted by two reviewers for quality assurance.

Data will be screened with the following five stages:

Stage One: electronic database search using terms; with results imported into reference management software, and duplicates removed.

Stage Two: title and abstract reviewed to remove studies not meeting the inclusion criteria (see above).

Stage Three: manuscript review to remove studies that did not meet inclusion criteria; paper selection.

Stage Four: review of references of selected papers (from Stage Three).

Stage Five: final paper selection, data extraction, and quality assessment.

Data will be extracted based on the specific points noted below and inputted into a standardised Excel database:

- study authors or agency, year
- study country
- setting: urban or rural
- population type (refugee; internally displaced; entrapped population; host population)
- humanitarian crises type (armed conflict or natural disaster)
- health outcome(s) addressed by the public health intervention
- type(s) of public health intervention
- study design
- target age group: i) infants: under 6 months; ii) infants and young children: under two ye ars; iii) children under five: 6 months - 59 months; iv) school age children: 6 years - 15 years; v) adolescents: 10 years - 19 years;* vi) adults: 20 years - 49 years; vii) elderly: 50+ years
- quality of the evidence on specific interventions
- change in quantity of evidence over time
- change in quality of evidence over time
- research strengths from the literature
- research gaps from the literature
- measurement outputs for quantitative data (e.g. confirmed use and/or self-reported use of services, etc)
- major themes in determinants of service utilisation from qualitative data

2.5 Quantitative data categorisation and analysis

Data findings will be organised in relation to the key issues of quantity and quality of the evidence base. To increase clarity of the final results, the studies selected at Stage Five will be arranged into three main categories of evidence (**Error! Reference source not found.**):

Table 1: Categorisation of selected literature

**Category A:**<br><br>Studies that measure statistical associations between intervention and health-related outcome
**Category B:**<br><br>Studies that measure changes in health-related outcome, but do not report statistical associations.
**Category C:**<br><br>Outcomes not measured (e.g. outputs, processes, perceptions)

As indicated in **Error! Reference source not found.**, Categories A and B roughly correspond to evidence that is expected to be of high to moderate quality. Given the generally much weaker value of evidence in Category C, data extracted from studies classified as Category C was limited to the existence of the study alone.

The quality assessment of quantitative studies (Categories A and B) that will be included in the systematic literature review will be reviewed based upon criteria adapted from the STROBE and CONSORT standards for observational studies and clinical trials, respectively. The adaptations are outlined in **Error! Reference source not found.** and scoring levels given in **Error! Reference source not found.**.

Table 2: Quality review criteria for quantitative studies (adapted from STROBE and CONSORT)

**STROBE Criteria for Observational Studies***	**CONSORT Criteria for Clinical Trials***
Intervention:<br>1. Is the intervention clearly described?<br>Selection of participants:<br>2. Is the target population defined?<br>3. Is there a comparison group (e.g. baseline, control)?<br>4. Are the inclusion and exclusion criteria defined?<br>Statistical methods:<br>5. Is the sample size / method justified with statistical basis?<br>6. Is there a statistical test (p-value or confidence interval)?<br>7. Is there adjustment for confounding?<br>Limitations:<br>8. Are study limitations explained (e.g. biases)?	Eligibility<br>1. Did study state # not meeting inclusion criteria?<br>2. Did study state # declined to participate?<br>Once Randomized:<br>Allocation:<br>3. Did study state # receiving intervention?<br>4. Did study state # not receiving intervention?<br>Follow-Up:<br>5. Did study state # lost to follow-up?<br>6. Did study provide reasons for loss to follow-up?<br>Analysis:<br>7. Did study state reasons participants were excluded from analysis?<br>8. Are limitations of the study explained (e.g. biases)

Table 3: Quality assessment corresponding to adapted STROBE and CONSORT criteria for quantitative studies

**Level of Quality**	**Rating of Evidence per STROBE / CONSORT**
HIGH	7-8 criteria met = **high** quality evidence
MODERATE	4-6 criteria met = **moderate** quality evidence
LOW	1-3 criteria met = **low** quality evidence

2.6 *Qualitative data categorisation and analysis*

To assess the determinants of service utilisation, a comparative thematic analysis will be conducted through the meta-analysis of common themes that emerge (eg. Population targeting, costs, knowledge of services, perceived quality of services through provider/client satisfaction). Although some themes will be identified a priori, the analysis will be guided by emerging findings. The CASP Qualitative Appraisal checklist will be used to determine the quality of qualitative studies.

The quality assessment of qualitative studies that will be included in the systematic literature review. The adaptations are outlined in Table 4 and scoring levels given in Table 5.

Table 4: Quality review criteria for qualitative studies (adapted from CASP)

CASP Checklist for Qualitative Studies	Yes	No	Uncertain
Research design:<br>1. Was the research design appropriate to address the aims of the research?<br>2. Was the data collected in a way that addressed the research issue?<br>Selection of participants:<br>3. Was the recruitment strategy appropriate to the aims of the research?<br>Ethical considerations:<br>4. Has the relationship between researcher and participants been adequately considered?<br>5. Have ethical issues been taken into consideration?<br>Analysis:<br>6. Was the data analysis sufficiently rigorous?<br>7. Is there a clear statement of findings?<br>8. How valuable is the research?			

Table 4: Quality assessment corresponding to adapted CASP criteria for qualitative studies

Level of Quality	Rating of Evidence per STROBE / CONSORT
HIGH	7-8 criteria met = **high** quality evidence
MODERATE	4-6 criteria met = **moderate** quality evidence

LOW	1-3 criteria met = **low** quality evidence

3. Search terms used for key bibliographic databases

(see Section 4 and 5 for specific search terms related to the effectiveness and service utilisation of SRH interventions which were added on to these)

1 exp Disasters

2 exp Relief Work

3 Rescue Work

4 Emergencies

5 Emergency Medicine

6 Emergency Medical Services

7 Disaster Medicine

8 Mass Casualty Incidents

9 Emergency Responders

10 Medical Missions, Official

11 (humanitarian adj2 (crisis or crises or relief or response or agenc$)).tw.

12 humanitarian.tw.

13 (disaster adj3 (relief or plan$)).tw.

14 ((relief or aid) adj2 work$).tw.

15 Refugees

16 (refugee or evacuee or evacuated).tw.

17 (displace$ adj2 (force$ or population or human or internal$)).tw.

18 Altruism

19 exp War

20 war.tw.

21 ((armed or zone) adj2 conflict$).tw.

22 (conflict affected adj3 (population$ or person$ or communit$)).tw.

23 Avalanches

24 Earthquakes

25 Floods

26 Landslide

27 Tidal Waves

28 Tsunamis

29 Cyclonic Storms

30 (typhoon$ or hurricane$ or cyclone$).tw.

31 (avalanche$ or earthquake$ or flood or floods or flooding or flooded or landslide$ or tsunami$).tw.

32 (disaster adj2 (natural or victim)).tw.

33 Droughts

34 drought$.tw.

35 Starvation

36 (starvation or famine$).tw.

37 or/1-36

38 randomized controlled trial

39 controlled clinical trial

40 cross-sectional studies

41 case-control studies

42 cohort studies

43 pilot studies

44 (random$ or controlled).tw.

45 (control adj3 (area or cohort? or compare? or condition or design or group? or intervention? or participant? or study)).ab. not (controlled clinical trial or randomized controlled trial).pt.

46 ((evaluat$ or prospective or retrospective) adj1 study).tw.

47 ("quasi-experiment$" or quasiexperiment$ or "quasi random$" or quasirandom$ or "quasi control$" or quasicontrol$ or ((quasi$ or experimental) adj3 (method$ or study or trial or design$))).tw.

48 ("time series" adj2 interrupt$).tw.

49 (intervention$ or impact or effectiveness or efficacy or service$ or outcome$ or output or treatment$ or management or program$ or project$).tw.

50 ((("semi-structured" or semistructured or unstructured or informal or "in-depth" or indepth or "face-to-face" or structured or guide) adj3 (interview* or discussion* or questionnaire*))).ti,ab. or (focus group* or qualitative or ethnograph* or fieldwork or "field work" or "key informant").ti,ab. or interviews as topic/ or focus groups/ or narration/ or qualitative research/

51 economics

52 cost-benefit analysis

53 cost control

54 Cost savings

55 cost of illness

56 cost $utility.tw.

57 (Cost$ adj2 effective$).tw.

58 cost-effective$.tw.

59 (cost adj3 utility).tw.

60 cost-utilit$.tw.

61 or/38-60

62 developing countries

63 exp asia

64 exp africa

65 exp pacific islands

66 exp eastern europe

67 exp china

68 balkan peninsula/ or europe, eastern/ or transcaucasia

69 caribbean region/ or central america/ or "gulf of mexico"/ or latin america/ or south america

70 atlantic islands/ or indian ocean islands/ or macau/ or pacific islands/ or philippines/ or prince edward island/ or svalbard/ or west indies

71 or/62-70

72 Japan

73 71 not 72

74 37 and 61 and 73

75 limit 74 to yr="1980 -2017”

4. Search terms specific to the <u>Utilisation</u> of SRH interventions including the MISP

Sources:

Published literature: Embase, Global Health, Medline, International Bibliography of the Social Sciences (IBSS), PsychINFO and Web of Science.

Sexual and Reproductive Health

"Reproductive Health"[Mesh] OR "Reproductive Medicine"[Mesh] OR "Sexual Dysfunction, Physiological"[Mesh] OR "Sexual Dysfunctions, Psychological"[Mesh] OR "Libido"[Mesh] OR "Sexology"[Mesh] OR "Sex Education"[Mesh] OR "Sexual Behavior"[Mesh] OR "Sexual Behaviour"[Mesh] OR "Sexuality"[Mesh] OR "Sexual Partners"[Mesh] OR "Sexual Minorities"[Mesh] OR "Sex Workers"[Mesh] OR "Sexual Health"[TIAB] OR "sexual health"[Mesh] OR "Sexuality"[TIAB] OR "Sexual Dysfunction"[TIAB] OR "Libido"[TIAB] OR "Sex Education"[TIAB] OR "Sexuality Education"[TIAB] OR "Sexology"[TIAB] OR "Sex Counselling"[TIAB] OR "Sex Counseling" [TIAB] OR "Sexual Behavio#r" [TIAB] OR "Sexual Partner*"[TIAB] OR "Sexual Minorit*"[TIAB] OR "Minimum Initial Service Package"[TIAB]

Contraception

"Reproductive plan*"[tiab] OR "contracepti*"[tiab] OR "contraceptive use"[tiab] OR "birth control"[tiab] OR "birth spacing"[tiab] OR "child spacing"[tiab] OR "condom*"[tiab] OR "the pill"[tiab] OR "oral contraceptive"[tiab] OR "oral contraceptives"[tiab] OR "microbicide"[tiab] OR "diaphragm"[tiab] OR "IUD"[tiab] OR "Intrauterine device"[TiAB] OR "Intrauterine Devices, Medicated"[Mesh] OR "Intrauterine Devices, Copper"[Mesh] OR "Intrauterine Devices"[Mesh] OR "Drug implant*"[MeSH] OR "Drug implant*"[tiab] OR "Drug Pellet*"[tiab] OR "Levonorgestrel"[MeSH] OR "Norethindrone"[MeSH] OR "contraceptive implant*"[tiab] OR "progestogen only contraceptive*"[tiab] OR "progestogen implant*"[tiab] OR "etonogestrel implants"[tiab] OR "Implanon"[tiab] OR "Subdermal contraceptive implant*"[tiab] OR "Norplant" [tiab] OR "Jadelle"[tiab] OR "Sino-implant"[tiab] OR "Depo Provera"[tiab] OR "Nexplanon"[tiab] OR "Norprogesterones"[tiab] OR "natural family planning"[tiab] OR "lactational amenorrhea" [tiab] OR OR "LAM"[tiab]" OR "postpartum amenorrhea"[tiab] OR "post-partum amenorrhea"[tiab] OR "amenorrhea"[mesh] OR "periodic abstinence"[tiab] OR "rhythm method"[tiab] OR "calendar method"[tiab] OR "sexual abstinence"[tiab] OR "Family Planning Services" [MeSH] OR "Reproductive Health Services"[MeSH] OR "Reproductive Health Services"[tiab] OR "Reproductive Medicine"[MeSH] OR "Contraception"[MeSH] OR "Contraception, Postcoital"[MeSH] OR "Contraception, Immunologic"[MeSH] OR "Contraception, Barrier"[MeSH] OR "Contraception Behaviour"[MeSH] OR "Natural Family Planning Methods"[MeSH] OR "Contraceptive Devices, Female[MeSH]" OR "Contraceptive Agents, Female"[MeSH] OR "Condoms, Female"[MeSH] OR "Sterilization, Reproductive"[MeSH] OR "family planning" [tiab] OR ("family planning" [Tiab] AND service* [TiAB]) OR ("family planning" [Tiab] AND program* [TiAB]) OR "Family Planning Services" [MeSH] OR "Family Planning Service"[tiab] OR "Family Planning"[MeSH] OR "Family Planning Programs"[Mesh] OR "Family Planning Program"[tiab] OR "population control"[mesh]

NOT Animals [mesh] NOT "Animal experimentation" [Mesh] NOT "Models, Animal" [Mesh]

Emergency Contraception:

"Contraceptives, Oral/administration and dosage"[Mesh] OR "Contraceptives, Oral/metabolism"[Mesh] OR "Contraceptives, Oral/pharmacology"[Mesh] OR "Contraceptives, Oral/physiology"[Mesh] OR "Contraceptives, Oral/therapeutic use"[Mesh]) AND "Contraceptives, Postcoital/administration and dosage"[Mesh] OR "Contraceptives, Postcoital/adverse effects"[Mesh] OR "Contraceptives, Postcoital/contraindications"[Mesh] OR "Contraceptives, Postcoital/metabolism"[Mesh] OR "Contraceptives, Postcoital/pharmacokinetics"[Mesh] OR "Contraceptives, Postcoital/standards"[Mesh] OR "Contraceptives, Postcoital/supply and distribution"[Mesh] OR "Contraceptives, Postcoital/therapeutic use"[Mesh] OR "ulipristal acetate"[Supplementary Concept]

Medical abortion

"Abortion, Induced"[Mesh] OR "Abortion, Incomplete"[Mesh] OR "Abortion, Spontaneous"[Mesh] OR “abortion” [tiab] OR “miscarriage”[tiab] OR “pregnancy termination”[tiab] OR “termination of pregnancy”[tiab] OR “abortal"[Tiab] OR "postabortion"[Tiab] OR "post-abortal"[Tiab] OR “postabortion care”[tiab] OR “incomplete abortion*”[tiab] OR "Mifepristone"[Mesh] OR "Misoprostol"[Mesh] OR “RU486”[tiab] OR “mifegyne”[tiab] OR “Cytotec”[tiab] OR “Medabon”[tiab] OR “medication abortion”[tiab] OR “medical abortion”[tiab] OR "unsafe abortion"[Tiab] OR "unsafe abortions"[Tiab] OR (("aftercare"[Tiab] OR "after care"[Tiab]) OR "aftercare"[Tiab]) OR ("postoperative"[Tiab] OR "post-operative"[Tiab]) AND "abortion"[Tiab])

Surgical abortion

"Abortion, Induced"[Mesh] OR "Abortion, Incomplete"[Mesh] OR "Abortion, Spontaneous"[Mesh] OR “abortion” [tiab] OR “miscarriage”[tiab] OR “pregnancy termination”[tiab] OR “termination of pregnancy”[tiab] OR “postabortion care” [tiab] OR “incomplete abortion”[tiab] OR "Extraction, Obstetrical"[Mesh] OR "Dilatation and Curettage"[Mesh] OR "Vacuum Curettage"[Mesh] OR “surgical abortion” [tiab] OR “dilation and evacuation”[tiab] OR “D&E”[tiab] OR “suction curettage”[tiab] OR “vacuum aspiration”[tiab] OR “D&C”[tiab] OR “menstrual regulation”[tiab]

Abortion-related complications

"Hemorrhage"[Mesh] OR "Postoperative Hemorrhage"[Mesh] OR "Uterine Hemorrhage"[Mesh] OR "Postpartum Hemorrhage"[Mesh] OR "Infection"[Mesh] OR "Pelvic Infection"[Mesh] OR "Uterine Perforation"[Mesh] OR "Uterine Rupture"[Mesh] OR "Pregnancy Complications"[Mesh] OR "Postoperative Complications"[Mesh] OR "Intraoperative Complications"[Mesh] OR "Emergency Treatment"[Mesh] OR “Abortion, Septic” [Mesh] OR “haemorrhage” [tiab] OR “haemorrhage”[tiab] OR “hemorrhage”[tiab] OR “bleeding”[tiab] OR “endometritis”[tiab] OR “parametritis”[tiab] OR “metritis” [tiab] OR “pelvic infection” [tiab] OR “uterine infection” [tiab] OR “uterine perforation” [tiab] OR “abortion-related complications”[tiab] OR “emergency care” [tiab] OR “ongoing pregnancy” [tiab] OR “ectopic pregnancy”[tiab] OR “emergency treatment” [tiab] OR “EmOC”[tiab] OR “emergency obstetric care”[tiab] OR “complications”[tiab] or “stillb*”[tiab] OR “birth”[tiab]

HIV/AIDS

“HIV”[Mesh] OR “AIDS”[tiab] OR “HIV/AIDS”[tiab] OR “Human Immunodeficiency Virus”[Mesh] OR “Human Immune Deficiency Virus”[tiab] OR “Acquired Immunodeficiency Syndrome”[Mesh] OR “Acquired Immune Deficiency Syndrome”[tiab] OR HIV [MesH] OR HIV-1 [MesH] OR HIV-2 [MesH] OR “HIV infections” [MesH] OR “acquired immunodeficiency syndrome” [MesH] OR “HIV seropositivity” [MesH] OR “HIV seroprevalence” [MesH] OR “AIDS serodiagnosis” [MesH]

STIs

“Chlamydia infections”[Mesh] OR “Gonorrhea”[Mesh] OR “chlamydia”[tiab] OR “gonorrhoea”[tiab] OR “syphilis”[tiab] OR “syphilis”[Mesh] OR “sexually transmitted infections”[Mesh] OR “sexually transmitted infection$”[tiab] OR “sexually transmitted disease$”[tiab] OR “sexually transmitted disease$”[Mesh] OR “hepatitis”[tiab] OR “chancroid”[tiab] OR “trichomoniasis”[tiab] OR “human papillomavirus”[tiab] OR “HPV”[tiab] OR “genital wart$”[tiab] OR “herpes”[tiab] OR “bacterial vaginosis”[tiab] OR “scabies”[tiab] OR “public lice”[tiab] OR “crab lice”[tiab] OR “pelvic inflammatory disease”[tiab] OR “PID”[tiab] OR “mucopurulent cervicitis”[tiab] OR “MPC”[tiab] OR “molluscum contagiosum”[tiab] OR “lymphogranuloma venereum”[tiab] OR “LGV”[tiab]

PMTCT

“antenatal HIV test”[tiab] OR “maternal HIV test”[tiab] OR “infant HIV test”[tiab] OR “infant HIV diagnosis”[tiab] OR “option A”[tiab] OR “option B”[tiab] OR “option B+”[tiab] OR “nevirapine”[tiab] OR “mother-to-child transmission”[tiab] OR “maternal transmission”[Mesh] “maternal transmission”[tiab] OR MTCT[tiab] OR “prevention of mother-to-child transmission”[tiab] OR “PMTCT”[tiab] OR “PMTCT cascade”[tiab]

Pregnancy, Maternal and Newborn Health

“Pregnan*"[Mesh] OR "Pregnancy, Unplanned"[Mesh] OR “"Pregnancy, unwanted"[Mesh] OR “Pregnancy in adolescence”[Mesh] OR “Pregnancy outcome”[Mesh] OR “Pregnancy, complication*”[Mesh] OR “Pregnan*”[tiab] OR “IUP”[tiab] OR “Intrauterine pregnancy”[tiab] “Pregnancy, complication*”[tiab] OR “Maternal health” [Mesh] OR “Maternal health” [tiab] OR “Maternal welfare” [Mesh] OR “Maternal welfare” [tiab]OR “Safe motherhood”[Mesh] OR “Safe motherhood”[tiab] OR “Perinatal”[Mesh] OR “Perinatal”[tiab] OR “Perinatal care”[Mesh] OR “Perinatal health”[Mesh] OR “Prenatal care”[Mesh] OR “Prenatal health”[Mesh] OR “Prenatal diagnosis”[mesh] OR “Perinatal care”[tiab] OR “Perinatal health”[tiab] OR “Postnatal health”[Mesh] OR “Antenat*”[Mesh] OR “Antenatal health”[Mesh] OR “Antenatal*”[tiab] OR “Ante-natal*”[tiab] OR “Prenatal*”[Mesh] OR “Prenatal*”[tiab] OR “postnatal care”[tiab] OR “postnatal”[tiab] OR “post-natal”[tiab] OR “Postpart*”[Mesh] OR “Post-part*” [Mesh] OR “Parturition”[Mesh] OR “Postpartum period”[MeSH] OR “Postpartum”[tiab] OR “Post-partum”[tiab] OR “Puerperium”[tiab] OR “Gestation”[tiab] OR “Postbirth”[tiab] OR “Post-birth”[tiab] OR “Mother*”[Mesh] OR “Matern*”[Mesh] OR “Childbirth”[tiab] or “Obstetrics”[tiab] OR “obstetric*”[Mesh] OR “Gynecology”[Mesh] OR “Labor pain”[Mesh] OR “Analgesia, Obstetric” [Mesh] OR “Obstetric Surgical Procedures” [Mesh] OR “Delivery, Obstetric”[Mesh] OR “Safe delivery”[Mesh] OR “Safe delivery”[tiab] OR “Skilled birth attend*”[Mesh] OR “Skilled birth attend*”[tiab] OR “Maternal

Health Service*" [Mesh] OR "Emergency Obstetric Care"[tiab] OR "Emergency Obstetric and Newborn Care"[tiab] OR "EmOC"[tiab] OR "EmONC"[tiab] OR "Infant"[Mesh] OR "Neonat*"[Mesh] "Neonatal health"[Mesh] OR "Infant health"[Mesh] or "Newborn health"[Mesh] OR "Newborn infant health"[mesh] OR "Infant welfare"[Mesh] OR "Baby health"[Mesh] OR "Newborn"[Mesh] OR "Infant, Newborn" [Mesh] OR "Birth*"[Mesh] OR "Fetus"[mesh] OR "Fetal therapies"[mesh] OR "Fetal monitoring"[mesh] OR "Infant"[tiab] OR "Neonat*"[tiab] "Neonatal health"[tiab] OR "Infant health"[tiab] OR "newborn health"[tiab] OR "Newborn infant health"[tiab] OR "Infant welfare"[tiab] OR "Baby health"[tiab] OR "Newborn"[tiab] OR "Stillbirth"[Mesh] OR "Still-birth"[Mesh] OR "Stillbirth"[tiab] OR "Still-birth"[tiab]

Vaginal Injury & Fistulas
"fistula"[Mesh] OR "fistula"[tiab] OR "rectovaginal fistula"[tiab] OR "rectovaginal fistula"[Mesh] OR "vaginal fistula"[Mesh] OR "urethra fistula"[tiab] OR "urinary tract fistula"[tiab] OR "genital trauma"[tiab] or "genital injury"[tiab] or "vaginal trauma"[tiab] or "vaginal injury"[tiab]

Adolescent Health
"adolescent sexual health"[tiab] OR "adolescent reproductive health"[tiab]OR "adolescent health"[tiab] OR "adolescent health services"[tiab] OR "youth friendly services"[tiab] OR "adolescent friendly services"[tiab] OR "adolescent health service"[tiab] OR "youth friendly service"[tiab] OR "adolescent friendly service"[tiab] OR "youth program*"[tiab]

Gender-based violence
"gender-based violence"[tiab] OR "partner violence"[tiab] OR "family violence"[tiab] OR "violence against women"[tiab] OR "domestic violence"[tiab] OR "sexual abuse"[Mesh] OR "sexual abuse"[tiab] OR "sex crime"[tiab] OR "sexual crime"[tiab] OR "domestic violence"[tiab] OR "domestic violence"[Mesh] OR "family violence"[Mesh] or "sexual violence"[tiab] OR "sexual violence"[Mesh] OR "rape"[tiab] OR "physical violence"[tiab] OR "rape"[Mesh] OR "intimate partner violence"[tiab] OR "intimate partner violence"[Mesh] OR "partner violence"[tiab] OR "partner abuse"[tiab] OR "spousal abuse"[tiab] OR "spouse abuse"[Mesh] OR "wife abuse"[tiab] OR "partner violence"[tiab] OR "partner violence"[Mesh] OR "assault"[tiab] OR "physical assault"[tiab] OR "sexual assault"[tiab] OR "sexual crime"[tiab] OR "sexual harassment"[Mesh] OR "sexual harassment"[tiab] OR "sexual coercion"[tiab] OR "forced sex"[tiab] OR "sexual slavery"[tiab] OR "abused woman"[tiab] OR "abused women"[tiab] OR "battered woman"[tiab] OR "battered women"[tiab] OR "battered women"[Mesh] OR "woman, abused"[tiab] OR "woman, battered"[tiab] OR "women, abused"[tiab] OR "women, battered"[tiab]

5. Search terms specific to service utilisation of SRH interventions including the MISP

Sources:

Published literature: Embase, Global Health, Medline, International Bibliography of the Social Sciences (IBSS), PsychINFO and Web of Science.

“use-effectiveness”[mesh] OR “health services accessibility”[Mesh] OR “sustained use”[Mesh] OR “uptake”[Mesh] OR “up-take”[Mesh] OR “utilization”[Mesh] OR “service utilisation”[Mesh] OR “patient acceptance of health care”[Mesh] OR “use-of-service”[tiab] OR “use of service”[tiab] OR “service use”[tiab] OR “effective use” [tiab] OR “sustained use”[tiab] OR “uptake”[tiab] OR “up-take”[tiab] OR “utilisation”[tiab] OR “service utilisation”[tiab] OR “health care utilisation”[tiab] OR “access”[tiab]
